# 4-Phenylbutyric acid mitigates ER stress-induced neurodegeneration in the spinal cords of a GM2 gangliosidosis mouse model

**DOI:** 10.1093/hmg/ddae153

**Published:** 2024-11-12

**Authors:** Fiona E Weaver, Elizabeth White, Allyson M Peek, Colin A Nurse, Richard C Austin, Suleiman A Igdoura

**Affiliations:** Department of Biology, McMaster University, 1280 Main St. W., Hamilton, ON L8S 4K1, Canada; Department of Biology, McMaster University, 1280 Main St. W., Hamilton, ON L8S 4K1, Canada; Department of Biology, McMaster University, 1280 Main St. W., Hamilton, ON L8S 4K1, Canada; Department of Biology, McMaster University, 1280 Main St. W., Hamilton, ON L8S 4K1, Canada; Department of Medicine, Division of Nephrology, McMaster University, 1280 Main Street W., Hamilton, ON, L8S 4L8, Canada; The Research Institute of St. Joe’s Hamilton and The Hamilton Center for Kidney Research, 50 Charlton Avenue E., Hamilton, ON, L8N 4A6, Canada; Department of Biology, McMaster University, 1280 Main St. W., Hamilton, ON L8S 4K1, Canada; Department of Pathology and Molecular Medicine, McMaster University, 1200 Main Street W., Hamilton, ON, L8S 4K1, Canada

**Keywords:** Tay Sachs, spinal cord, ER stress, lysosome, lysosomal storage disease

## Abstract

Sandhoff disease (SD), a fatal and rare lysosomal storage disorder (LSD), is caused by a deficiency of the enzyme β-hexosaminidase B and leads to severe accumulation of GM2 gangliosides in lysosomes, primarily within the central nervous system (CNS). This accumulation results in severe neurological impairment, lower motor neuron disease, and death. Currently, there are no effective therapies available for SD. Here, we explored the role of endoplasmic reticulum (ER) stress in the spinal cord during disease progression in an established mouse model of SD and revealed the beneficial outcome of off-label treatment with the FDA-approved drug, 4-phenylbutyric acid (4-PBA). We analyzed the expression and localization of ER stress and cellular apoptosis markers, which revealed significant upregulation of these factors within motor neurons. Additionally, we observed a > 50% reduction in neuronal numbers throughout all spinal cord regions. Our studies also tested the impact of the chemical chaperone 4-PBA on ER stress in mice, and following administration, we observed significant improvements in motor neuromuscular function and life span throughout disease progression. 4-PBA treatment significantly reduced apoptosis in spinal cord neurons and increased the number of choline acetyltransferase (ChAT)-positive neurons, with little effect on astrogliosis or sensory interneurons. Overall, this study provides strong evidence for the role of chronic ER stress in the pathophysiology of SD and highlights 4-PBA as a promising therapeutic treatment for SD and potentially other related LSDs.

## Introduction

GM2 gangliosidoses are a group of neurodegenerative lysosomal storage disorders (LSDs) that include Tay-Sachs disease (TSD) and Sandhoff disease (SD). These disorders have similar phenotypic presentation, and the severity/onset of disease depends on the pathogenic variants involved and residual enzymatic activity. SD is a result of severe GM2 and GA2 accumulation within the CNS due to variants in the HEXB gene, which encodes the β subunit of the lysosomal enzymes β-hexosaminidase A and B [1, 2]. For both TSD and SD, disease onset falls within three categories: infantile, juvenile, and late-onset. Symptomology for these disorders is considered a consequence of spinal cord dysfunction, including a multitude of neuromuscular deficits, such as tremors, muscle weakness, ataxia, and severe cognitive impairment [1–3]. Neurons are a primary site for ganglioside synthesis, making them particularly vulnerable to these diseases, and the loss of motor neuron functionality correlates with the phenotypic characteristics of these disorders. Late-onset SD (LOSD) is frequently misdiagnosed as a lower motor neuron disorder such as amyotrophic lateral sclerosis (ALS), multiple sclerosis (MS), or spinal muscular atrophy (SMA) [4–6], with motor neuron disease symptoms being reported in 42% of LOSD patients [7]. In fact, within the anterior horn of the spinal cord, where motor neurons are located, diffuse neuronal degeneration and enlarged neurons have been reported repeatedly in LOSD, and this pathophysiology closely resembles that of lower motor neuron diseases.

Assessment of neurodegenerative diseases and their underlying mechanisms is highly dependent on the availability of animal models. For GM2 gangliosidoses, *Hexb^−/−^* mice are a well-characterized model for both TSD and SD, portraying the severe phenotypes observed in humans. This model displays the characteristic motor neuromuscular dysfunction, behavioral deficits, mass neurodegeneration, and early death, reaching an endpoint by ~120 days of age. Studies using this model have established the role of apoptosis in neurodegeneration; however, the mechanisms connecting lysosomal storage with programmed cell death remain to be elucidated.

Multiple disease mechanisms for LSDs have been proposed, including dysfunctional mitochondria, severe neuroinflammation, lysosomal rupture, impaired autophagy, and calcium (Ca^2+^) dysregulation [1, 8–13]. However, another potential disease mechanism that has received little attention is endoplasmic reticulum (ER) stress and the activation of the unfolded protein response (UPR), both of which are highly interconnected with the previously mentioned mechanisms [14]. The ER is a dynamic organelle involved in protein folding and processing, lipid biosynthesis, and Ca^2+^ homeostasis, and as such, is a central regulator of cellular homeostasis. Due to its multifaceted nature, the ER directly interacts with other organelles, including the plasma membrane, mitochondria, peroxisomes, and Golgi apparatus via membrane contact sites [15–18]. A continuity between lysosomes and the ER has been demonstrated which acts as a pipeline to facilitate the exchange of Ca^2+^ between lysosomes and the ER [19, 20]. However, in LSDs, dysfunctional lysosomes lose membrane integrity causing leakage and resulting in the syphoning of ER Ca^2+^, depletion of internal stores, and the induction of ER stress [21, 22]. Under homeostatic conditions, the resident ER chaperone protein glucose-regulated protein 78 (GRP78) is associated with the three transmembrane proteins of the UPR: activating transcription factor 6 (ATF6), protein kinase RNA-like endoplasmic reticulum kinase (PERK), and inositol requiring enzyme 1 (IRE1), keeping them inactive. However, during ER stress, GRP78 is recruited to assist with the growing accumulation of misfolded proteins, thereby relieving its inhibition of the three signaling factors [23]. Interestingly, GRP78 can alternately localize to the cell surface, where it has been demonstrated to impact cell survival and proliferation [24–26]. Furthermore, activation of the UPR triggers a cascade of downstream effector molecules, such as the potent transcription factor X-box binding protein 1 (XBP1), the proapoptotic factor C/EBP homologous protein (CHOP), and the executioner caspase, cleaved caspase 7, which can lead to apoptosis [27].

Preliminary mRNA evaluation of multiple ER stress factors, including ATF6, GRP78, and XBP1, revealed ER stress induction in cultured fibroblasts from several neurodegenerative and non-neurodegenerative LSDs [28]. In a mouse model of GM1 gangliosidosis, Tessitore et al. described increased mRNA levels of CHOP, activated c-Jun N-terminal kinase 2 (JNK2), and caspase 12, along with increased TUNEL-positive neurons, which indicates UPR induction during disease progression [14]. Caspase activation has also been reported in other LSDs, such as mucopolysaccharidosis type II (MPS II), where patient-derived TD35 neuronal cultures showed significantly increased levels of caspase 3 and 7 [29]. ER stress induction is a highly dynamic process in which many of its factors undergo posttranslational modifications and translocations, alluding to the importance of evaluating ER stress and the UPR more thoroughly at the cellular level. Furthermore, revealing underlying disease mechanisms opens the door for novel therapeutic modalities, such as the utilization of chemical chaperones, including sodium phenylbutyric acid (4-PBA), which enhances protein folding and stability of GRP78 expression and, overall, obviates ER stress [30–34].

In this study, we utilized the *Hexb*^−/−^ mouse model to evaluate several UPR components during the pathophysiology of the spinal cord. The *Hexb*^−/−^ mouse model is considered one of the best neurodegenerative mouse models because it accurately recapitulates the pathophysiological aspects of SD, making it a valuable tool for investigating therapeutics. We present strong evidence that chronic ER stress occurs in SD mouse spinal cords throughout development, beginning as early as 60 days of age. Furthermore, we demonstrate that persistent UPR activation is associated with the nuclear localization of ATF6, CHOP and XBP1, along with caspase 7 activation and increased apoptotic events. Moreover, our findings clearly highlight the efficacy of oral administration of 4-PBA in alleviating ER stress activation, rescuing spinal motor neurons, and significantly increasing the lifespan of SD mice.

## Materials and methods

### Study design

The goal of this study was to elucidate the role of ER stress during SD progression and evaluate the therapeutic effect of modulating the ER stress response for the treatment of SD. Tissue samples collected from Hexb knockout (*Hexb^−/−^*) mice, at various ages, were examined for ER stress. Healthy littermates expressing WT Hexb (*Hexb^+/+^*) were used as controls. Indicators of ER stress induction were histological evaluation and cellular measurements. Furthermore, *Hexb^−/−^* mice were treated with chemical chaperone 4-phenylbutyric acid, again using healthy littermates (*Hexb^+/+^*) as controls. Therapeutic readouts following treatment with 4-PBA were histological analysis, cellular measurements, neuromuscular function tests, and survival. Experimental groups were sized to allow for statistical analysis; all animals were included in the analysis, and no outliers were excluded. Mice were assigned randomly to the treatment group, and the researcher who performed drug delivery and functional analyses was not blinded to group identity. Data collection was halted when mice met pre-determined endpoint criteria in accordance with guidelines provided by animal ethics. Functional tests were performed in triplicates, averaging the three trials to obtain results. For histological analyses, an n = 3 was used to assess immunostaining.

### Mice

Mouse work was conducted under the animal utilization protocol (AUP) in accordance with the Ontario Animals for Research Act specifications and the Animal Research Ethics Board (AREB) guidelines. The Sandhoff model mice (*Hexb^−/−^* on a C57BL/6 background) were previously characterized [35].

### Genotyping

Mice were genotyped using isolated tail genomic DNA as a template for PCR. For Hexb genotyping, 2 forward primers were used, one for wild type 5′-GGTTTCTACAAGAGACATCATGGC-3′ and one for knockout 5′-GATATTGCTGAAGAGCTTGGCGGC-3′, with a common reverse primer 5′-CAATCGGTGCTTACAGGTTTCATC-3′ to generate a 141 bp product for the wild-type allele and a 700 bp product for the knockout allele. The thermocycler program consisted of 35 cycles of 94°C for 30 s, 60°C for 30 s and 72°C for 45 s.

### Animal protocols

Protocol one: Two groups of *Hexb^−/−^* mice (n = 10 per group); one group was administered 4-PBA (Scandinavian Formulas, Inc.) 4 mg/ml (0.4% w/v) in drinking water at 40 days of age. Body weights and vital signs were monitored, and neuromuscular motor function was assessed every other day starting at 50 days of age until the endpoint. At the endpoint, the mice were harvested for tissue histology and protein analyses. Protocol Two: Two groups of *Hexb^−/−^* mice at 40 days of age (n = 4 per group) were established. One group was administered 4-PBA 4 mg/ml (0.4% w/v) in drinking water, and one group did not receive any 4-PBA. Mice were perfused at 80 days of age with formalin and processed for immunohistochemistry. Spine sections from untreated and treated mice were immunolabeled with multiple antibodies and evaluated.

### Behavioral testing

The motor neuromuscular impairment, body weight, and lifespan of each group of mice were monitored. Motor coordination and neurological integrity were evaluated utilizing wire hang and righting response behavioral tests, respectively, which were conducted every other day following 40 days of age as previously described [12, 36].

### Immunohistochemistry

*Hexb^+/+^* and *Hexb^−/−^* mice harvested at a range of ages, including 40, 60, 80, 100, and 120 days, were perfused with PBS and fixed with 10% buffered formalin. The spinal cord was harvested, sectioned into three main regions (cervical, thoracic, and lumbar), decalcified, and then processed and embedded in paraffin wax. Cross-sections of the spinal cord were cut at 4 μm and mounted on charged slides. Immunostaining was then performed on these samples using a Vectastain Elite ABC Universal Plus Kit (Vector Laboratories: PK-8200). Slides were rehydrated, first by washing slides with xylene followed by a 1:1 solution of xylene to ethanol (EtOH) and subsequently by washes in 100% EtOH, 95% EtOH, 70% EtOH, 70% EtOH/1% hydrogen peroxide solution, 70% EtOH/1% lithium carbonate solution, 70% EtOH, and finally, 50% EtOH. Next, antigen retrieval in sodium citrate buffer (pH 6) was performed by microwaving the slides at 1 min intervals until boiling and then maintaining a boil for 5 min. The slides were then washed with tap water followed by a wash in glycine. Next, the slides were incubated with Bloxall blocking solution for 10 min, followed by a 5 min wash with PBS. Slides were then blocked with 2.5% horse serum for 20 min. Primary antibodies for ER stress markers, including anti-GRP78 (1:200, Novus Biologicals: NBP1-06277), anti-ATF6 (1:50, Novus Biologicals: NBP1-40256), anti-XBP1 (1:100, Novus Biologicals: NBP1-77681), anti-GFAP (1:100, Sigma; G9269), anti-ChAT (1:200, Sigma, AMAB91130), anti-Calretinin (1:200, Proteintech, 12 278-1-AP) anti-CHOP (1:400, Novus Biologicals: NBP2-13172), anti-cleaved caspase 7 (1:400, cell signaling; 8438S), anti-NeuN (1:200, Novus Biologicals; NBP1-92693), and normal mouse serum (1:500, Santa Cruz Biotech; sc-2025) were used. Slides were incubated with antibodies overnight at 4°C. Slides were washed with 1x PBS + 0.05% TWEEN20 for 3 x 5 min prior to application of the secondary antibody. Slides were then incubated with prediluted biotinylated horse anti-mouse/anti-rabbit IgG secondary antibody for 30 min. Slides were then again washed with 1x PBS + 0.05% TWEEN20, and Vectastain elite ABC reagent was then added to the slides for 30 min and washed with 1x PBS. Equal amounts of ABC reagents 1 and 2 were mixed, and slides were incubated with the solution until staining appeared (~5–20 min) and then rinsed with tap water. Counterstaining was then completed by placing slides into 0.1% methylene blue for 1.5 min and removing excess with water. Slides were then washed in EtOH starting at 50% and going up to 100%, then 1:1 xylene:EtOH, and finally 100% xylene. Finally, glass slide covers were mounted using Permount histological mounting medium (Fisher Scientific: SP15-500). Images were captured at various magnifications via a Nikon Eclipse Ci microscope equipped with a Nikon DS-Ri2 camera.

### Immunofluorescence

Spinal cords from *Hexb^−/−^* and *Hexb^+/+^* mice were harvested at 40, 60, 80, 100, and 120 days of age and embedded in paraffin as described above. Samples were rehydrated, first by washing slides with xylene then by washing them with a 1:1 solution of xylene and ethanol (EtOH) followed by washes in 100% EtOH, 95% EtOH, 70% EtOH, 70% EtOH/1% lithium carbonate solution, 70% EtOH, and finally, 50% EtOH. Next, antigen retrieval in sodium citrate buffer (pH 6) was performed by microwaving the slides at 1 min intervals until boiling and then maintaining a boil for 5 min. The slides were then washed with tap water followed by a wash in glycine. Slides were then blocked with 2.5% horse serum for 20 min. Samples were then double labeled with primary antibodies and incubated overnight at 4°C. Samples were washed with 1x PBS + 0.05% TWEEN20 for 3 x 5 min prior to application of the secondary antibody. Next, slides were double labeled with appropriate Alexa Fluor secondary antibodies (Invitrogen, goat anti-rabbit 488: A11008, goat anti-mouse 594: A11029, goat anti-rabbit Alexa 594; A11037, goat anti-mouse Alexa 488; A10667) and then again washed with 1x PBS + 0.05% TWEEN20. Finally, the slides were mounted with ProLong Diamond antifade mounting reagent with DAPI (Invitrogen: P36966). Samples were imaged using a Zeiss Axiovert 200 microscope equipped with an HBO 100 mercury lamp.

### Statistical analysis

For data sets with groups of two that needed to be compared, t tests were used to test for differences between means at *P* < 0.05. Data sets of three or more groups were tested for differences between means at *P* < 0.05 using one-way ANOVA. This was followed by Tukey’s post hoc test for all data sets with equal variance and high normality between groups. For sample sets with unequal sample sizes or variance, the Kruskal–Wallis test was used for pairwise comparisons. All statistical analyses were performed using GraphPad Prism 9 (V.9.3.0).

## Results

### Histological abnormalities and neuronal loss in the spinal cords of *Hexb ^−/−^* mice

**Figure 1:**
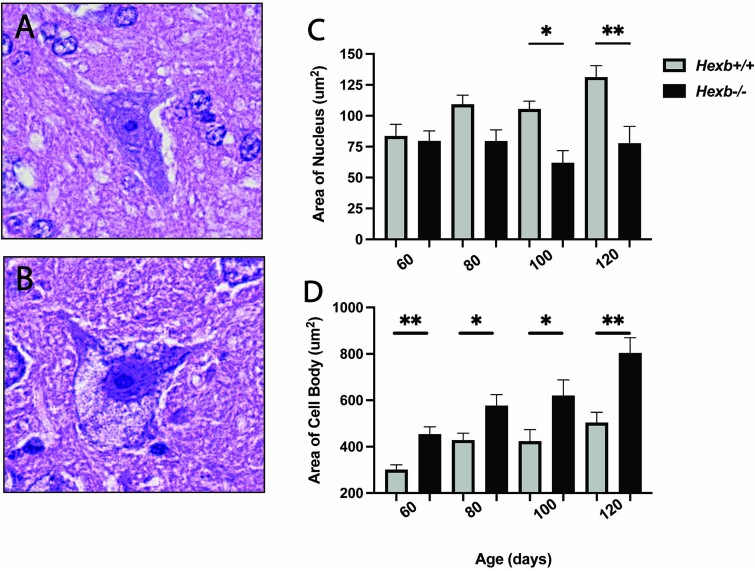
Morphological abnormalities in spinal neurons of SD mice. A quantitative analysis of neuronal morphological changes seen in the spinal cord motor neurons (arrowhead) of *Hexb^+/+^* and *Hexb^−/−^* mice during the pathogenesis of SD. Representative images of a single anterior horn motor neuron from *Hexb^+/+^* (A) and *Hexb^−/−^* (B) spinal cords. Quantification of nuclear area (C) and cell body area (D) in *Hexb^+/+^* and *Hexb^−/−^* spinal cord sections. Cell body area n = 20, nuclear area n = 20. ^*^P < 0.05, ^*^^*^P < 0.0022 error bars ±SEM.

**Figure 2:**
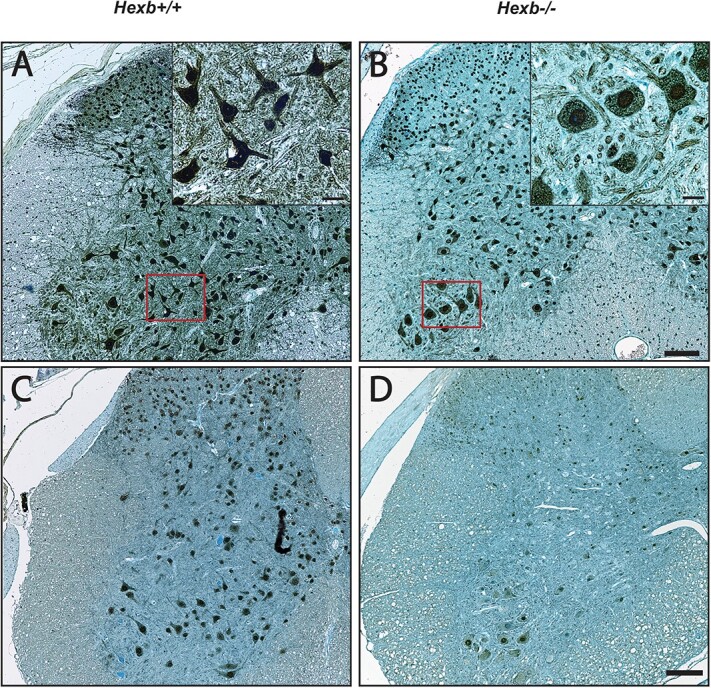
Mass neuronal loss throughout the spinal cord of SD mice at terminal disease stage. Representative immunohistological micrographs showing the neuronal-specific marker NeuN in the cervical region of the spinal cords of *Hexb^+/+^* (A and C) and *Hexb^−/−^* (B and D) mice. (A and B) High-magnification images of anterior horn motor neurons of 40-d-old *Hexb^+/+^* and *Hexb^−/−^* mice. Scale bar = 10 μm. (C and D) Low-magnification images of NeuN immunoreactivity in AHNs from 120-day-old *Hexb^+/+^* and *Hexb^−/−^* mice. Scale bar = 100 μm.

LSD progression causes significant morphological changes in the spinal motor neurons of *Hexb^−/−^* mice, such as engorged cell bodies, rugged cell membranes and shrunken nuclei, in comparison to the neurons of *Hexb^+/+^* mice (Fig. 1A and B). In fact, in SD mice, the average neuronal cell body area (μm^2^) was significantly increased, and that of the nucleus (μm^2^) was significantly reduced (Fig. 1C and D). Interestingly, anterior horn neurons (AHNs) in *Hexb^−/−^* mice appeared to have retracted their axonal projections starting as early as 40 days of age, which contrasted with the morphology of wild-type (WT) neurons (Fig. 2A and B). The overall number of NeuN-positive neurons within the anterior horns of *Hexb^−/−^* mice was found to gradually decrease over time compared to that of WT mice (Fig. 2C and D). At 120 days of age, *Hexb^−/−^* mice showed severe neuronal loss (>50%) within the various regions of the spinal cord compared to *Hexb^+/+^* mice. These histological and morphological differences accentuate cellular stress and neuronal damage due to lysosomal dysfunction within the spinal cords of *Hexb^−/−^* mice.

### GRP78 expression and its trans-localization to the cell surface of ANHs

**Figure 3:**
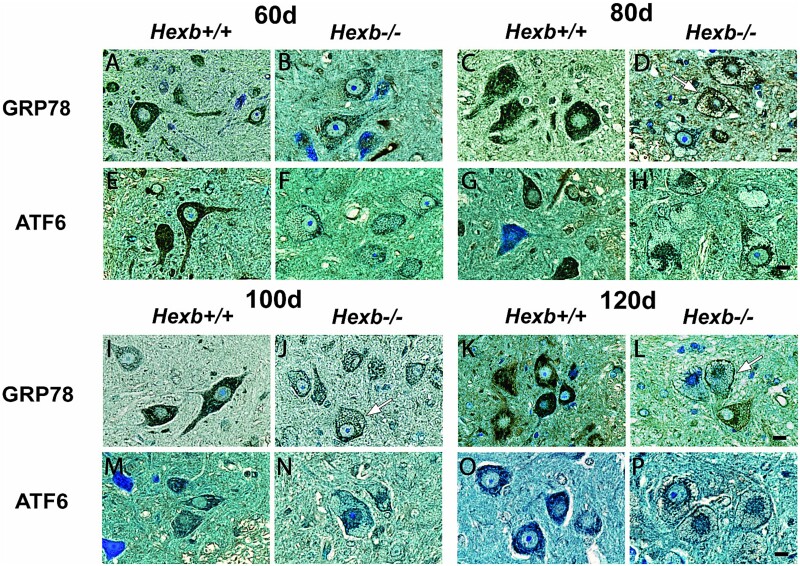
Presence and localization of major UPR markers within anterior horn neurons. Representative immunohistological micrographs of the localization of the ER stress markers GRP78 and ATF6 within anterior horn motor neurons in the spinal cords of 60-, 80-, 100- and 120-day-old *Hexb^+/+^* and *Hexb^−/−^* mice. Arrows indicate differential GRP78 localization to the cytosol and cell surface in *Hexb^−/−^* AHNs. Scale bar = 10 μm.

**Figure 4:**
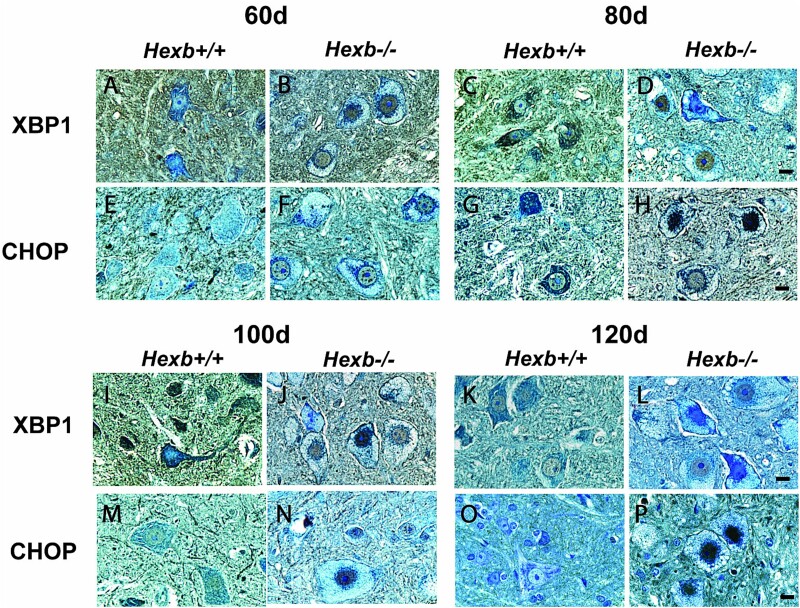
Nuclear localization of downstream UPR players XBP1 and CHOP in the anterior horn neurons of SD mice. Representative immunohistological micrographs of the ER stress markers XBP1 and CHOP within the cervical spinal cord region of 60-, 80-, and 100-day-old *Hexb^+/+^* and *Hexb^−/−^* mice. Scale bar = 10 μm.

To assess the role of ER stress in the motor neurons of *Hexb^−/−^* mice, we immunohistochemically examined the expression levels of UPR markers, namely, GRP78, ATF6 (Fig. 3), as well as XBP1, and CHOP (Fig. 4), in the spinal cords of *Hexb^+/+^* and *Hexb^−/−^* mice at different ages. Overall, spinal motor neurons displayed distinct temporal differences in the localization and expression of GRP78 between *Hexb^+/+^* and *Hexb^−/−^* mice. Typically, WT motor neurons, particularly AHNs, exhibited unique intense reticular GRP78 staining with distinct immunoreactive Nissl bodies that were consistent across various ages (Fig. 3A, C, I, and K). In contrast, GRP78 cellular localization and expression in *Hexb^−/−^* AHNs showed significant changes throughout disease progression (Fig. 3B, D, J, and L). Beginning at 60 days of age, *Hexb^−/−^* mice exhibited marginally lower reticular GRP78 immunoreactivity, followed by the tapering of intracellular GRP78 expression with age until 120 days (endpoint) was reached. In concert with the reduction in GRP78 expression, *Hexb^−/−^* AHNs also showed changes in their intracellular localization. Three unique patterns of localization were highlighted. First, AHNs from *Hexb^−/−^* mice showed diffuse cytosolic staining, which was drastically different than the punctate, reticular staining present in the *Hexb^+/+^* mice. Second, GRP78 localized to a halo surrounding the nuclear membrane, which was most notable at 80 and 120 days of age (Fig. 3D and L). Finally, GRP78 underwent trans-localization to the cell surface, beginning at 80 days and persisting until 120 days of age (Fig. 3D, J, and L). The cell surface expression of GRP78 is a hallmark feature of ER stress.

### Upregulation of ER stress markers (ATF6/XBP1) in *Hexb^−/−^* spinal cords

ATF6 immunoreactivity in *Hexb^+/+^* spinal cord sections was observed within the AHNs and dorsal horn as intense reticular staining (Fig. 3E, G, M, and O). Conversely, AHNs in the *Hexb^−/−^* spinal sections exhibited some contrasting ATF6 immunostaining that changed during disease development. A reticular pattern indicative of ER-bound or Golgi-retained ATF6 was observed within the 60-day-old *Hexb^−/−^* AHNs (Fig. 3F). Upon examination of 80-day-old *Hexb^−/−^* samples, a translocation of ATF6 in several of the AHNs from strictly reticular to partially nuclear was noted (Fig. 3H). Despite some staining remaining within the cytosol, the distribution of cytosolic immunolabeling changed from diffuse and punctate to clustered around the nucleus in an ER halo. Furthermore, the staining intensity of ATF6 was reduced in 80-day-old *Hexb^−/−^* samples in comparison to age-matched *Hexb^+/+^* sections. This signified that ATF6 expression tapers off in parallel with disease progression. Similarly, 100- and 120-day-old samples also showed a combination of ER halo and nuclear staining within AHNs (Fig. 3N and P). These results indicate a transient activation and translocation of ATF6 between 80–120 days of age.

To extend our investigation to downstream targets of ATF6 and IRE1, we examined the temporal expression of XBP1. Spinal cords from 60-day-old *Hexb^+/+^* mice revealed minimal levels of cytosolic immunoreactivity across many cell types in all three regions (Fig. 4A). A reduction in staining intensity was observed toward the posterior region of each section. The AHNs exhibited the heaviest cytosolic staining, with very few cells displaying weak nuclear staining. This staining pattern indicated the presence of inactive or uncleaved XBP1, which suggested inactivity of IRE1 and therefore a lack of ER stress and UPR activation during homeostatic conditions. Similar findings were noted in 80-, 100-, and 120-day-old *Hexb^+/+^* spinal cord samples (Fig. 4C, I, and K). In contrast, the *Hexb^−/−^* mice revealed differential staining patterns, localization, and expression levels at the various ages examined. In 60-day-old *Hexb^−/−^* mice, the same dispersal pattern was maintained in which staining decreased from the ventral horn to the dorsal horn. However, in sharp contrast, the *Hexb^−/−^* AHNs showed a striking translocation of XBP1 from the cytosol into the nucleus, most notably at 80 and 100 days of age (Fig. 4B, D, J, and L). XBP1 cleavage, which activates its transcriptional activity of UPR target genes, is completed after the induction of ER stress. This allows for the translocation of XBP1 to the nucleus, which was the localization pattern observed in the AHNs of *Hexb^−/−^* mice. Overall, activated ATF6 and XBP1, both with clear nuclear localization within the *Hexb^−/−^* spinal cord sections, provide further evidence that the UPR is highly activated during disease pathogenesis and that at least 2 of the 3 UPR signaling arms are involved.

### Early indications of chronic stress and neuronal apoptosis

The identification of activated ER stress markers such as GRP78, ATF6, and XBP1 as early as 60 days of age within the spinal cords of *Hexb^−/−^* mice raised the question of whether neuronal apoptosis occurs much earlier than the expected endpoint of 120 days of age. We therefore evaluated the expression and localization of CHOP, a pro-apoptotic transcription factor (Fig. 4E–H and M–P). CHOP is a key regulator in cell fate determination, as it plays a vital role in the apoptotic decision of cells. Cytosolic immunostaining of CHOP, predominantly within AHNs, was observed throughout the *Hexb^+/+^* spinal cords, with most other cell types void of CHOP immunoreactivity (Fig. 4E, G, M, and O). CHOP is ubiquitously expressed at low levels, and therefore, cytosolic expression is expected under homeostatic conditions. This localization pattern is observed consistently within the spinal cord across all ages of *Hexb^+/+^* mice. In contrast, *Hexb^−/−^* spinal sections demonstrated a striking trans-localization of CHOP to the nucleus. Interestingly, at 60 days of age, several AHNs already exhibited nuclear staining ranging from light to intense, and upon further evaluation, foci were noted in several of the stained nuclei (Fig. 4F). Similarly, 80-day-old *Hexb^−/−^* spinal sections demonstrated clear nuclear localization of CHOP with obvious intranuclear punctate staining (Fig. 4H). By 100 and 120 days of age, the AHNs showed clear morphological characteristics of the disease, and this was accompanied by heavy nuclear immunostaining of CHOP, as seen in earlier ages (Fig. 4N and P). Immunoreactivity was more concentrated within the anterior and posterior horns of spinal sections, with lesser amounts in the intermediate zone. Overall, these results support earlier findings which indicate ER stress and the UPR are induced during SD progression, but this also suggests that CHOP-mediated apoptotic pathways are being invoked in response to the disease.

### Alteration of ER stress induction following 4-PBA treatment

**Figure 5:**
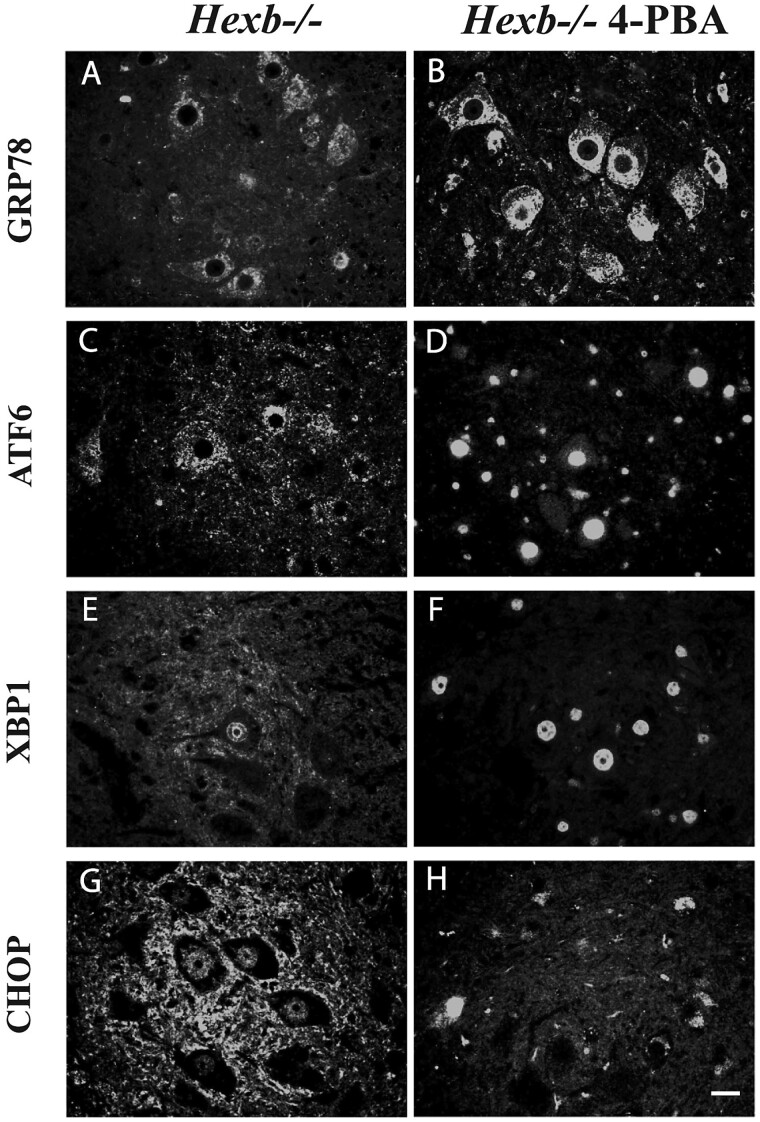
Treatment with chemical chaperone, 4-PBA, negatively regulates ER stress activation. Representative immunofluorescent labeling of GRP78, ATF6, XBP1, CHOP and phosphorylated eukaryotic factor 2 alpha-positive (p-eIF2α) in the spinal cord from *Hexb^−/−^* mice untreated or treated with 4-PBA. Note that *Hexb^−/−^* AHNs showed mild reticular staining of GRP78 and ATF6, sparse nuclear staining of XBP1 and intense reticular staining of p-eIF2⍺. While 4-PBA *Hexb^−/−^* AHNs showed increased GRP78, ATF6, and XBP1 staining, a shift in ATF6 from cytosolic to nuclear localization and a reduction in p-eIF2⍺ staining resulted in a significant reduction in the number of eIF2α-positive neurons with cytoplasmic staining after 4-PBA treatment. Bars represent 10 μm.

To evaluate the impact of ER stress attenuation, we administered the pharmacological chaperone 4-PBA in drinking water to *Hexb^−/−^* mice starting at 40 days of age. UPR activation was assessed through immunofluorescence staining of GRP78-, ATF6-, XBP1-, CHOP-, and phosphorylated eukaryotic initiation factor 2 alpha (p-eIF2α)-positive neurons in spinal cord sections from treated or untreated *Hexb^−/−^* mice (Fig. 5). Spinal samples from untreated *Hexb^−/−^* mice revealed that AHNs had sparse cytosolic and ER halo GRP78 staining, while 4-PBA-treated *Hexb^−/−^* mice showed intense, punctate reticular staining (Fig. 5A and B). ATF6 immunolabeling within *Hexb^−/−^* AHNs was primarily located in the cytosol (Fig. 5C). In sharp contrast, 4-PBA *Hexb^−/−^* AHNs had heavy ATF6 nuclear localization (Fig. 5D). Furthermore, untreated *Hexb^−/−^* AHNs revealed minimal XBP1 labeling, with a few AHNs showing nuclear localization (Fig. 5E). Following treatment with 4-PBA, a dramatic increase in the presence of XBP1 labeling was observed, and *Hexb^−/−^* AHNs showed marked nuclear localization (Fig. 5F). Immunolabeling of the apoptotic marker CHOP was observed in the nucleus of untreated *Hexb^−/−^* AHNs (Fig. 5G); however, treated *Hexb^−/−^* AHNs revealed differential localization and intensity (Fig. 5H). Following treatment, *Hexb^−/−^* AHNs had a reduction in CHOP immunoreactivity, and it was present in the cytosol, which contrasted with the nuclear staining seen in *Hexb^−/−^* AHNs. p-eIF2α staining showed variation between the anterior and posterior horns as well as between treated and untreated *Hexb^−/−^* mice (Fig. 5I–L). In untreated *Hexb^−/−^* mice, the cells in both the anterior horn and posterior horn showed cytosolic staining (Fig. 5I and K). However, following treatment with 4-PBA, a significant reduction in the number of cytosolic p-eIF2α-positive neurons was observed in the anterior horn, and within the posterior horn, with some neurons showing distinct nucleolar staining. (Fig. 5J and L). The phosphorylation of eIF2α demarcates the presence of ER stress, and therefore, the loss of cytosolic staining following treatment supports the function of 4-PBA in alleviating ER stress.

### Treatment with 4-PBA reduces apoptotic events within the spinal cord

**Figure 6:**
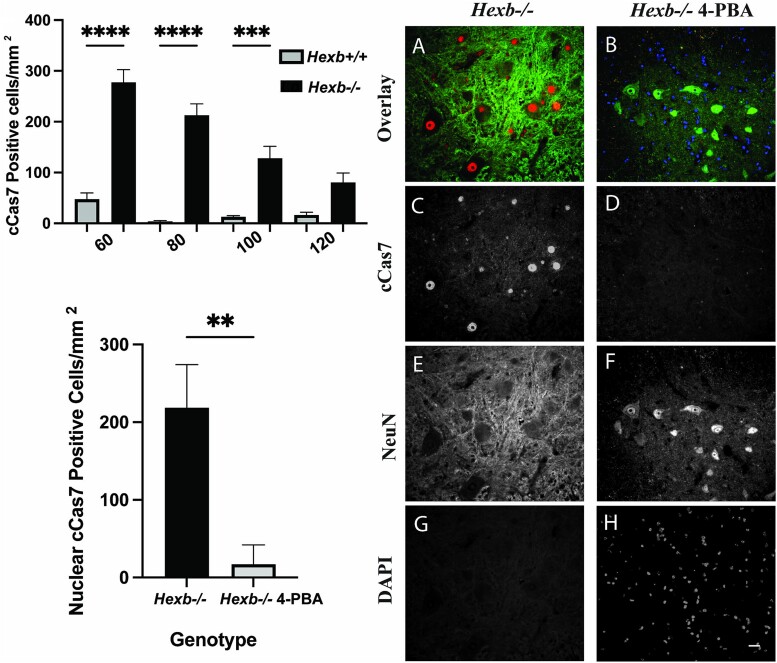
4-PBA successfully inhibits the nuclear localization of cleaved caspase 7 in anterior horn neurons of SD mice. (A) Quantification of the frequency of cleaved caspase 7-positive cells in the spinal cord of 60-, 80-, 100- and 120-day-old *Hexb^+/+^* and *Hexb^−/−^* mice. (B) Representative double-label immunofluorescence micrographs of cleaved caspase 7-positive neurons and the neuronal marker NeuN in the spinal cord from *Hexb^−/−^* mice untreated or treated with 4-PBA. Note the heavy nuclear localization of cCAS7 in untreated Hexb*^−/−^* AHNs and a significant reduction in the number of cCAS7-positive AHNs in 4-PBA-treated *Hexb^−/−^* AHNs. Bars represent 10 μm. (C) Quantification of the number of cleaved caspase 7-positive neurons in untreated and 4-PBA-treated *Hexb^−/−^* spinal cords.

Previously identified activation of two vital potentiators of ER stress during SD progression led us to evaluate the intracellular localization of the executioner caspase cleaved caspase 7 (cCas7), temporally in the spinal cords of *Hexb^+/+^* and *Hexb^−/−^* mice, to determine a timeline of the mass neuronal death known to be associated with SD pathology. To elucidate the differences in cCas7 expression and its temporal variation in localization between *Hexb^+/+^* and *Hexb^−/−^* mice, we quantified the number of nuclear cCas7-positive cells within the anterior horns of spinal cord samples. Within the cervical, thoracic, and lumbar regions, a predefined area of the anterior horn was established, the total number of cells that presented with strictly nuclear cCas7 staining was counted, and data were pooled to represent the totality of the spinal cord (Fig. 6A). Three independent sets of spinal cords were evaluated, and the numbers were standardized as cells/mm^2^. Interestingly, the peak of cCas7 activation was observed at 60 days of age, which was far earlier than previously hypothesized. Subsequent deterioration of cCas7-positive cell numbers was seen in a stepwise manner throughout the remaining ages. These observations clearly demonstrate the immense activation of cCas7 during early disease progression and provide evidence that this mechanism of apoptosis occurs much earlier than expected, leading to prominent neuronal degeneration in the SD mouse model. Furthermore, spinal cord sections were double immunolabeled for cCas7 and the neuronal marker NeuN. *Hexb^−/−^* spinal cord segments demonstrated intense nuclear localization of cCas7, which was an indication of its activation (Fig. 6B). Following treatment with 4-PBA, there was a 92% reduction in the number of cCas7-positive cells present in the anterior horn of the spinal cord (Fig. 6B and C). These results indicated a significant reduction in the number of cCas7-positive neurons present throughout the spinal cord following 4-PBA treatment. Overall, these results demonstrated that 4-PBA significantly decreases ER stress-induced cCas7 activation in SD pathophysiology.

### 4-PBA treatment rescues motor neurons in *Hexb^−/−^* spinal cords

**Figure 7:**
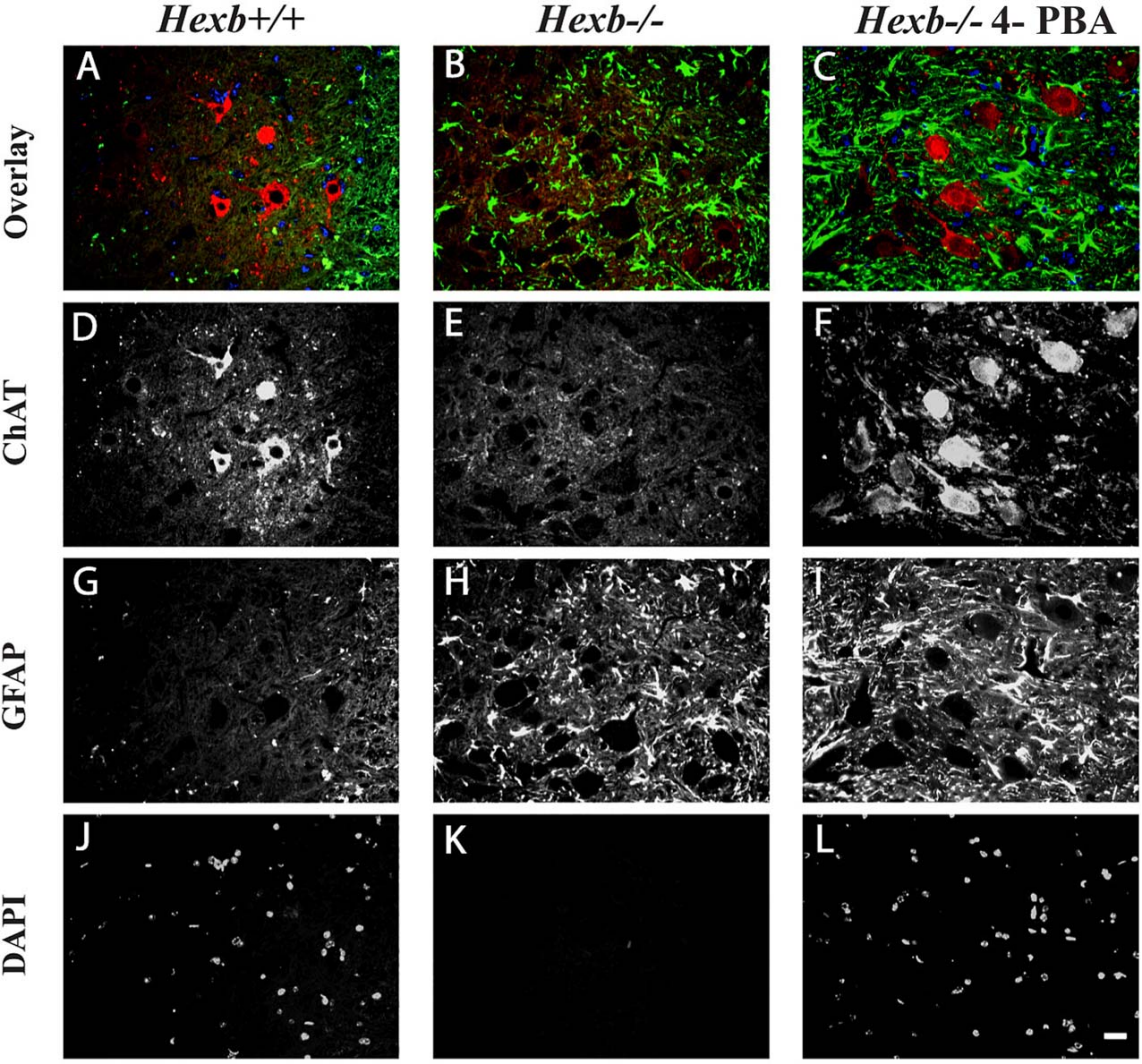
Administration of 4-PBA to SD mice results in retention of cholinergic motor neurons within the spinal cord. Representative immunofluorescence images of GFAP, ChAT and DAPI staining in the anterior horns of spine sections from *Hexb^+/+^*, *Hexb^−/−^*, and 4-PBA-treated *Hexb^−/−^* mice. Note intense cytosolic ChAT staining in AHNs of *Hexb^+/+^* mice diminished ChAT staining in *Hexb^−/−^* mice with active astrogliosis and intense cytosolic ChAT staining with active astrogliosis, as seen in 4-PBA-treated *Hexb^−/−^* AHNs. Bar represents 10 μm.

**Figure 8:**
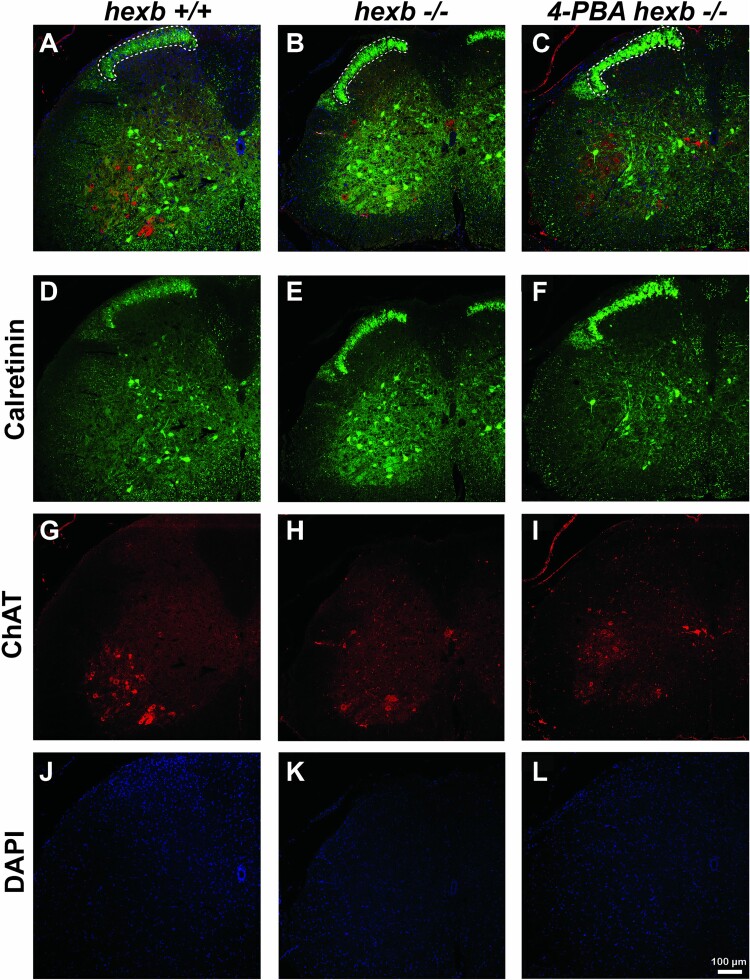
The presence of calretinin-positive interneurons is regulated by 4-PBA. Representative immunofluorescent labeling of Calretinin-positive interneurons (sensory) and ChAT-positive neurons (motor) in spinal cord sections from *Hexb^+/+^*, *Hexb^−/−^*, and 4-PBA-treated *Hexb^−/−^* mice. Note that ChAT-positive motor neurons were maintained in the anterior horn (AH, arrows) following 4-PBA treatment, while calretinin-positive interneurons densely stained the posterior horn (PH, arrowheads), with an increase in labeling intensity within the anterior horn of *Hexb^−/−^* spinal cord mice. Bars represent 100 μm.

ChAT (choline acetyltransferase) produces acetylcholine, an excitatory neurotransmitter in the autonomic nervous system that regulates muscle contractions, and other functions. Loss of acetylcholine has dire health issues, including seizures and muscle spasms. We evaluated motor neuron survival versus astrogliosis utilizing immunostaining for ChAT and GFAP. Immunolabelling in the anterior horn of spinal sections from *Hexb^+/+^*, *Hexb^−/−^* and 4-PBA treated *Hexb*^−/−^ mice showed intense cytosolic ChAT (a marker for cholinergic motor neurons) staining in AHNs of *Hexb^+/+^* while diminished ChAT staining and increased GFAP staining (a glial cell marker, indicative of active astrogliosis) observed in *Hexb^−/−^* mice. In addition, sections from 4-PBA treated *Hexb^−/−^* show intense cytosolic ChAT staining within viable AHNs and intense GFAP staining (Fig. 7). Our observation indicates that while 4-PBA potentiates AHNs survival, it has little effect on active astrogliosis within the spinal cord of *Hexb^−/−^* mice. Secondly, we evaluated the frequency of ChAT-positive neurons versus calretinin-positive interneurons (sensory processing neurons) in 80-day old mice. Our results highlighted a significant loss of motor neurons but the survival of sensory interneurons throughout each segment of the spinal cord of SD mice (Fig. 8). Immunolabelling with calretinin and ChAT in spinal cord sections of untreated and 4-PBA treated *Hexb^−/−^* mice revealed that ChAT-positive motor neurons were maintained in the anterior horn following 4-PBA treatment, while calretinin-positive interneurons densely stained the posterior horn, with an increase in labeling intensity within the anterior horn of *Hexb^−/−^* spinal cord sections (Fig. 8). Of particular interest, C-boutons can be noted within the WT spinal samples, while in the *Hexb^−/−^* mice, there is a mass reduction in numbers, and their localization is very dispersed. However, following treatment with 4-PBA, ChAT staining was normalized, if not more intense, with C-boutons prevalent surrounding motor neurons throughout the anterior horn (Fig. 8).

### Chemical chaperone 4-PBA impacts neuronal size and muscle mass

**Figure 9:**
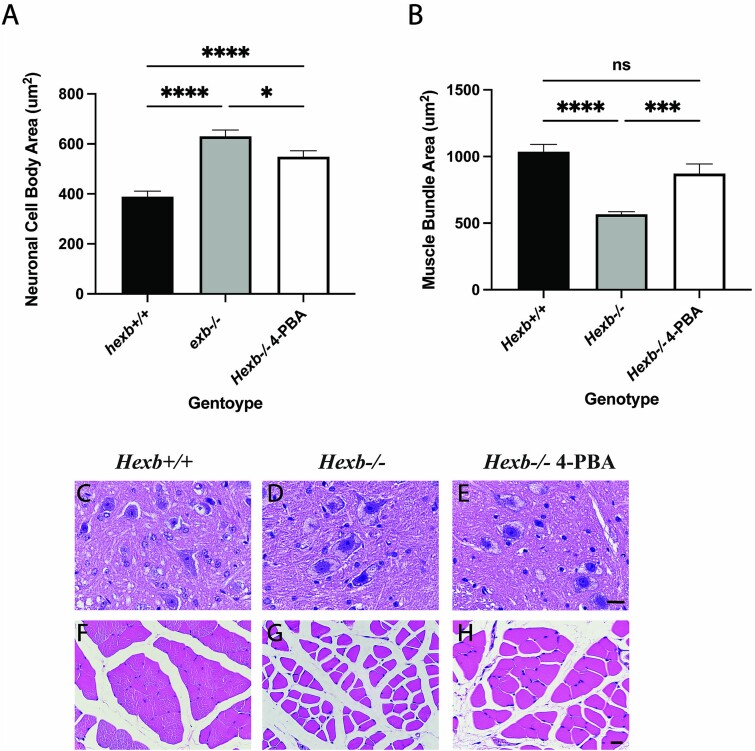
4-PBA improves neuronal and muscle fiber morphological abnormalities in SD mice. (A) Cell body sectional area of the anterior horn neurons and (B) the cross-sectional area of muscle fibers from the muscles surrounding the spinal cord of 80-day-old *Hexb^+/+^* and *Hexb^−/−^* mice. Histological sections of spinal cord (C–E) and skeletal muscle (F–H) from *Hexb^+/+^*, *Hexb^−/−^*, and 4-PBA-treated *Hexb^−/−^* mice. Within *Hexb^+/+^* mice, muscle fibers had an average area of ~ 1000 μm^2^, while in contrast, *Hexb^−/−^* mice showed a 44% reduction in area, with an average of ~ 570 μm^2^. 4-PBA treatment of *Hexb^−/−^* mice maintained normal cross-sectional areas of muscle myofibrils, which is consistent with functional cholinergic neurons.

Neuromuscular deficits are a highly prevalent consequence of SD. Therefore, we evaluated the cell body sectional area of AHNs and the cross-sectional area of muscle fibers from the muscles surrounding the spinal cord of 80-day-old *Hexb^+/+^* and *Hexb^−/−^* mice (Fig. 9). Within *Hexb^+/+^* mice, muscle fibers had an average area of ^~^1000 μm^2^; in contrast, *Hexb^−/−^* mice showed a 44% reduction in area with an average of ^~^570 μm^2^. Such a drastic reduction early in disease pathology indicates that muscle atrophy and neuronal loss had already occurred. Following treatment with 4-PBA, the muscle fiber cross-sectional area was found to be within the *Hexb^+/+^* range, averaging ~900 μm^2^, with no significant differences noted between the *Hexb^+/+^* and 4-PBA-treated *Hexb^−/−^* mouse muscle fiber cross-sectional areas (Fig. 9B). A significant difference was observed in the skeletal muscles of untreated and treated *Hexb^−/−^* mice, indicating that 4-PBA maintains neuromuscular innervation and reduces muscle atrophy.

### 4-PBA significantly improves motor function of *Hexb^−/−^* mice

**Figure 10:**
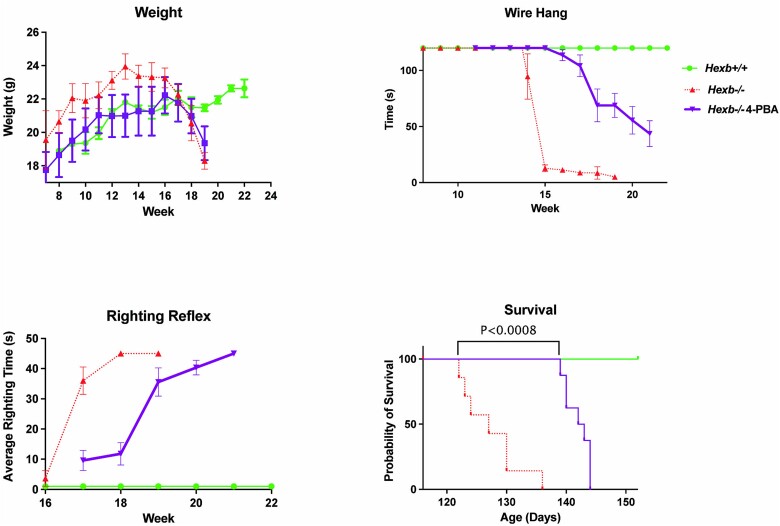
Significant improvements in motor neuromuscular function and lifespan following treatment with 4-PBA. Motor neuron assessment of *Hexb^+/+^* and *Hexb^−/−^* mice with and without 4-PBA. Mice underwent behavior testing as early as 7 weeks of age to measure degeneration of various cerebellar and peripheral nervous system functions. Measures of body mass (A), wire hang (B), righting reflex (C) and life span (D) were recorded over time. Measurements suggest that 4-PBA has a beneficial effect on SD. *Hexb^−/−^* mice on 4-PBA live significantly longer than mice without 4-PBA and display a significantly faster righting reflex and longer wire hang during later stages of disease, indicating a positive effect on general motor functions. In addition, *Hexb^−/−^* mice treated with 4-PBA have a similar increase in body mass as *Hexb^+/+^* mice. Analysis of survival by mantel-Cox test, *P* < 0.0001 between *Hexb^−/−^* mice without 4-PBA and *Hexb^−/−^* mice with 4-PBA, median survival = 128 days and 143 days, respectively.

To assess the physiological efficacy of 4-PBA on overall motor neuromuscular function, *Hexb^−/−^* mice treated with 4-PBA underwent behavioral testing beginning at 7 weeks of age. Utilizing a variety of physiological tests such as wire hang, body weight, and righting response, measures of degeneration of cerebellar and motor neuromuscular functions were collected (Fig. 10). Analysis of body mass measurements revealed that treatment with 4-PBA restored the weights of *Hexb^−/−^* mice to levels comparable to those of WT mice until 18 weeks of age when it declined similarly to the untreated *Hexb^−/−^* mice. (Fig. 10A). Additionally, examination of motor neural impairment utilizing a wire hang test revealed a significant increase in the wire hang time of treated mice in comparison to untreated mice (Fig. 10B). 4-PBA-treated mice showed a more gradual decline in performance, which is indicative of delayed neuromuscular impairment. Furthermore, assessing balance and motor coordination by examining righting response times highlighted that treatment with 4-PBA resulted in a significantly faster righting response within *Hexb^−/−^* mice (Fig. 10C). Untreated mice suffered a massive increase in righting response time by 17 weeks of age, while in contrast, treated mice demonstrated a 2-week delay, with faster times being recorded until 19 weeks of age. Finally, an analysis of survivability was conducted using a Mantel-Cox test (Fig. 10D). 4-PBA had a beneficial impact on lifespan, with a significant difference (*P* < 0.0001) in survival noted between treated and untreated SD mice. Overall, an increase of 16 days was noted, with 128 days as the median survival age for untreated *Hexb^−/−^* mice and 143 days for 4-PBA-treated *Hexb^−/−^* mice. These results indicate that 4-PBA dramatically reduces the severity of neuromuscular degeneration in *Hexb^−/−^* mice, thereby supporting its therapeutic potential for the treatment of SD.

## Discussion

In GM2 gangliosidosis, the connection between lipid accumulation within lysosomes and neuronal cell death is still not completely understood. However, our examination of ER stress provides a potential pathway connecting enzymatic deficiency and lysosomal storage to apoptotic outcomes. These results highlight a multitude of ER stress markers that are differentially expressed temporally throughout all regions of the spinal cord. They also uncover great diversity in the interregional distribution patterns, striking changes in cellular localization, neuronal cell loss, morphological changes, and apoptosis.

The ER has been at the center of several studies that have attempted to highlight a mechanistic pathway within several LSDs. Pelled and colleagues (2003) examined the implications of GM2 accumulation in neuronal tissue collected from *Hexb^−/−^* mice. They were able to identify a causal relationship between the intercellular accumulation of GM2 and rates of Ca^2+^ uptake via the sarco/endoplasmic reticulum Ca^2+^ ATPase (SERCA). In microsomes collected from *Hexb^−/−^* mouse brains, severely diminished Ca^2+^ uptake via SERCA into the ER was noted [9]. Depleted internal ER Ca^2+^ stores, which disrupt Ca^2+^ homeostasis, are a major inducer of ER stress. Other research has also suggested that the depletion of ER Ca^2+^ stores and activation of PERK can be induced by GM2 accumulation within neurons [37]. These results support the notion of ER stress induction following GM2 accumulation; however, to replicate storage conditions of the disease *in vitro,* the cells were exposed to high concentrations (2 μM) of exogenous GM2 [37]. Gangliosides cluster at the cell membrane, where they are capable of interacting with membrane proteins to modulate a variety of cellular functions, such as cell–cell recognition, phenotypic changes, cell growth, and signal transduction [38, 39]. When ganglioside levels are altered, they can lead to reduced membrane fluidity, which negatively impacts the cell and generates cellular dysfunction [40]. In these studies, the exogenous addition of GM2 creates an alteration in ganglioside levels and may result in cellular stress independent of substrate accumulation. However, our results demonstrate, *in vivo*, the implications of ER stress in SD progression as a result of GM2 accumulation and provide evidence that ER stress is a hallmark of disease pathophysiology.

Similar studies have been conducted in which primary neurons were isolated from a different LSD mouse model to evaluate the effects of substrate accumulation. The D’Azzo group determined that in spinal neurons, ER Ca^2+^ was disrupted and that there was an upregulation of CHOP because of GM1 accumulation [14]. This research, as well as the results from our study, provides strong evidence for ER stress involvement when ganglioside accumulation occurs within neurons. A separate study by Kobolak and colleagues utilized a disease-specific induced pluripotent stem cell (iPSC) line representative of mucopolysaccharidosis II (MPS II) to evaluate disease-related neuropathy [29]. These cells were differentiated into neuronal progenitor cells (NPCs) and then terminally differentiated toward cortical neurons. Although this study did not specifically assess the role of ER stress, they were able to identify an increase in the expression of cCas 7, an executioner for apoptotic events, within the diseased cell line compared to the control [29]. Therefore, these results provided evidence of increased apoptotic activity occurring within neurons of LSD patients. Incidentally, several studies have reported cCas7 upregulation in ALS patients, which could be used to extrapolate a potential common mediator of lower motor neuron disease symptoms seen between these disorders [41, 42].

Many UPR factors play a multitude of roles during the initiation and subsequent activation of downstream cascades depending on a variety of factors, such as the length and intensity of ER stress as well as the cell type involved [43]. The role of ATF6 is still not completely understood, but most research surrounding it has delved into its role as a pro-survival factor. ATF6 has been observed to exhibit a pro-survival role in response to chronic, mild ER stress [44]. Other studies have demonstrated a vital protective role through targeted downregulation of ATF6, which results in increased melanoma cellular sensitivity to the ER stress inducers: thapsigargin and tunicamycin [44, 45]. One study has demonstrated that ATF6 is a pro-survival factor that works to counteract ER stress activation by upregulating ER chaperones, such as GRP78 and XBP1 [46]. In contrast, some studies have also highlighted the roles of ATF6 in the apoptotic events induced by ER stress. In vascular endothelial cells, high expression of activated ATF6 was observed to exacerbate ER stress-induced apoptosis [43]. Conversely, ATF6 has been shown to activate and upregulate the expression of a multiplicity of apoptosis-invoking factors, such as CHOP and cleaved caspase 9, but it can also trigger inflammation through the induction of the NF-κB pathway [43, 47, 48]. Ultimately, our current results question the true role of ATF6 in SD and whether it activates pro-survival or pro-apoptotic signaling. At 60 days, we observed retention of ATF6 within the ER/Golgi of *Hexb^−/−^* AHNs, which suggests incomplete activation of ATF6, as well as a marginal reduction in expression compared to the *Hexb^+/+^* samples. In contrast, by 80 days, we observed a further decrease in overall expression as well as the presence of nuclear localization within some cells. However, following treatment with 4-PBA, ATF6 expression was enhanced and observed strictly within the nucleus. Therefore, whether the role of ATF6 is pro-survival or pro-apoptotic is uncertain; however, within our model, ATF6 appears to play a role in neuronal survival. When it is downregulated and minimally activated in the untreated SD mice, the frequency of apoptotic events is high, as noted by the presence of cCas7. Previous reports have documented the importance of ATF6, and how preventing its activation can induce ER stress and the UPR response, resulting in cellular apoptosis [46, 49, 50]. Knocking out ATF6 in cells decreased their tolerance to stress and resulted in persistent expression of CHOP and downregulation of ER chaperones, which are similar outcomes to what we observed in SD mice [51]. Further studies will need to be conducted to fully dissect the role of ATF6 in disease pathophysiology.

Concurrent with these ATF6 alterations, specifically between treated and untreated SD mice, we also noted changes in the expression of XBP1, a downstream target of the IRE1α pathway. 4-PBA-treated SD mice demonstrated a dramatic increase in XBP1 expression, with heavy nuclear labeling observed. This contrasted with untreated mice, which showed mild nuclear expression. This variation in expression may be a result of ATF6 activation. ATF6 controls the expression of XBP1, while IRE1α controls its activation. With ATF6 activation suppressed during SD pathogenesis, XBP1 upregulation would also be hindered. However, when ATF6 translocates to the nucleus following treatment, XBP1 upregulation occurs. Heavy nuclear XBP1 staining not only demonstrates the activation of ATF6 but also highlights that IRE1α is functional. Both arms have been predicted to support the pro-survival pathway of the UPR. During chronic ER stress, a decrease in IRE1α and ATF6 expression has been previously described, and by artificially maintaining these signaling pathways, cell survival was promoted and, in some cases, allowed for cellular escape from chemically induced ER stress [52]. The mechanistic switch from pro-survival to pro-apoptosis during ER stress has previously been linked to attenuation of the IRE1α and ATF6 signaling pathways, while the PERK pathway and its downstream apoptotic markers (CHOP) are maintained [52]. This attenuation was noted in our untreated SD mice; however, following treatment, recovery of these signaling pathways was observed. The maintenance of the ATF6 and IRE1α pathways was accompanied by motor neuron survival and mass decreases in apoptotic events, further highlighting their pro-survival role in SD disease pathology.

The notion that GRP78 becomes upregulated during ER stress to assist with the accumulation of misfolded proteins is widely accepted. Accompanying this upregulation, GRP78 has also been noted to undergo translocation to the cytosol, the nucleus, and most interestingly, the cell membrane [25]. Cell surface GRP78 has been tightly linked to ER stress, where it has been reported to participate in a wide range of signaling pathways, influencing processes such as proliferation, inflammation, and apoptosis [53–55]. With its role at the cell membrane being diverse and highly contextual, it can either confer cellular benefits or consequences [24, 43, 46, 56]. Our results demonstrated that within the spinal cords of SD mice, neurons exhibited localization of GRP78 to the cell surface early in disease progression, starting at approximately 80 days of age. The translocation of GRP78 to the cell surface is described as cell context-dependent but has been previously shown to occur in neurons that had undergone stress, including oxygen–glucose deprivation [56]. Furthermore, this redistribution was accompanied by a tapering of GRP78 expression in parallel with disease pathogenesis. However, this could be a result of its intracellular translocation or changes in global ER morphology, which can negatively impact GRP78 functionality and allow for chronic UPR activation. GRP78 has also been previously reported to interact with pro-caspase 7, suppressing its activation and revealing an antiapoptotic role for this ER chaperone protein [24, 54]. Downregulation of GRP78 has been correlated with an increase in the expression of multiple caspases, including caspase 7 [57]. Within our model, it could be suggested that a downregulation in GRP78 during SD and its subsequent redistribution throughout the cell, such as to the cell membrane, could result in a diminished ability to bind caspase 7, therefore allowing for the activation of apoptotic cascades. Our research provides data that support this notion in that we observed a reduction in GRP78 expression in *Hexb^−/−^* samples beginning at 60 days, which aligns with the peak of nuclear cCas7-positive cells in the anterior horn. However, it should be noted that there are other potential mechanisms that could result in the consequential activation of caspase 7, independent of ER stress factors [58]. Furthermore, following treatment with 4-PBA, SD mouse spinal cords demonstrated a massive increase in GRP78 expression while still maintaining GRP78 localization to the cell membrane, cytosol, and ER. 4-PBA functions to stabilize the expression of heat shock proteins, such as GRP78; however, we speculate that its upregulation may be due to a downstream effect in which the strong induction of ATF6 and XBP1 noted in the treated SD mice results in the upregulation of GRP78. Increased expression of this ER chaperone has been reported to protect cells from apoptosis through mechanisms such as inhibition of caspase 7 [24, 59]. These results highlight a unique response of GRP78 during SD pathogenesis, but how GRP78 influences disease progression, the pathway responsible for GRP78 upregulation, and its relationship with caspase 7 will require further research to understand fully.

Here, we have uncovered a hallmark mechanistic pathway involved in SD development and progression and provide promising data identifying several factors as potential therapeutic targets. We further confirmed this mechanism of ER stress by testing the efficacy of the chemical chaperone 4-PBA in alleviating ER stress and reducing neurodegeneration in SD. 4-PBA not only works as a chemical chaperone to assist in reducing the accumulation of misfolded proteins within the ER but also functions as an inhibitor of histone deacetylases to stabilize the expression of heat shock proteins [33]. This chemical chaperone is currently FDA-approved for urea cycle disorders and has been shown to mitigate disease pathology in other neurodegenerative disorders, such as ALS and SMA [60–62]. Within our model, not only did 4-PBA result in the upregulation of factors such as ATF6, XBP1, and GRP78, but we also noted a prominent reduction in the frequency of apoptotic events seen by the lack of cCas7 in treated SD spinal cords. These changes ultimately translated to the survival of neurons, more specifically motor neurons, within SD spinal cords.

Alpha motor neurons are the final common pathway for all motor commands, and once they are damaged or lost, there is no alternative route available to convey the information. Large cholinergic inputs, known as C-boutons, selectively innervate these alpha motor neurons, modulating their activity [63, 64]. C-boutons are found in higher abundance on motor neurons that innervate large proximal muscles compared to those that innervate small distal muscles [65]. Alterations in C-bouton density have been described in spinal cord dysfunction and disease. Following spinal cord injury, decreased motor neuron excitability was observed which correlated with reduced C-bouton abundance. However, in ALS, neuronal excitotoxicity was reported with enlarged C-boutons present throughout the spinal cord [65]. We reported a dramatic reduction in C-bouton abundance during SD, which highlights a loss of motor neurons, as well as a decrease in innervation to remaining neurons, leading to severe muscle atrophy. In sharp contrast, following treatment with 4-PBA, the presence of C-boutons and motor neurons was markedly increased, indicating that treatment allowed for the retention and protection of these synapses, which translates into the preservation of motor neurons and promotion of their survival. From here, a variety of potential therapeutic interventions can be explored, such as combinations with other chemical chaperones, including tauroursodeoxycholic acid (TUDCA), which target varying aspects of the disease, such as apoptosis. Another possibility is combining 4-PBA with already-approved treatments, such as substrate reduction therapy, utilizing drugs including Migalastat, thereby targeting both the initial insult of ganglioside accumulation and the main mechanism responsible for neuronal death.

In summary, we have highlighted significant changes in the morphological features of AHNs, as well as severe neuronal loss of ~50% in the spinal cord by the terminal stages of SD in *Hexb^−/−^* mice. We also established differential immunohistochemical localization and expression of ATF6, XBP1, GRP78, CHOP, and cCas7 between *Hexb^−/−^* and *Hexb^+/+^* spinal cord sections. We provided evidence demonstrating that ER stress induction and apoptotic events occur considerably earlier in the timeline of SD progression, approximately 80 days, compared to the original prediction of onset of 100–120 days. The ER is emerging as a vital regulator of cell fate and apoptosis, and these findings present ER stress and the UPR, as well as subsequent activation of potent inducers of apoptosis, as novel mechanistic pathways involved in SD pathogenesis. We further validated this mechanism by demonstrating the efficacy of the chemical chaperone 4-PBA in treating neuronal degeneration in SD mice. This treatment resulted in an upregulation of GRP78 and pro-survival UPR pathways, a decrease in the frequency of apoptotic events, and most importantly enhanced motor neuron survival. SD and TSD, while differing in underlying enzymatic deficiencies, present virtually identical phenotypes in humans. Our results therefore may also be highly relevant to the more common TSD, and there may be possibilities to further extend these discoveries to various types of LSDs, as the current literature indicates similarities between their underlying disease mechanisms. Therefore, the relevancy of these results may extend far beyond the current disease of interest, which would allow for significant advances in the mechanistic unraveling of several detrimental diseases and therapeutic innovations. Overall, our study highlights ER stress, the UPR, and consequential apoptosis as a pathway of interest and the efficiency of 4-PBA to alleviate ER stress and combat neurodegeneration in Sandhoff disease.
